# Applications of the SR4G Transgenic Zebrafish Line for Biomonitoring of Stress-Disrupting Compounds: A Proof-of-Concept Study

**DOI:** 10.3389/fendo.2021.727777

**Published:** 2021-11-17

**Authors:** Amin Nozari, Selena Do, Vance L. Trudeau

**Affiliations:** Department of Biology, University of Ottawa, Ottawa, ON, Canada

**Keywords:** zebrafish, endocrine-disrupting compounds, biomonitoring assay, environmental toxicology, transgenic model, stress-axis

## Abstract

Transgenic zebrafish models have been successfully used in biomonitoring and risk assessment studies of environmental pollutants, including xenoestrogens, pesticides, and heavy metals. We employed zebrafish larva (transgenic SR4G line) with a cortisol-inducible green fluorescence protein reporter (eGFP) as a model to detect stress responses upon exposure to compounds with environmental impact, including bisphenol A (BPA), vinclozolin (VIN), and fluoxetine (FLX). Cortisol, fluorescence signal, and mRNA levels of eGFP and 11 targeted genes were measured in a homogenized pool of zebrafish larvae, with six experimental replicates for each endpoint. Eleven targeted genes were selected according to their association with stress-axis and immediate early response class of genes. Hydrocortisone (CORT)and dexamethasone (DEX) were used as positive and negative controls, respectively. All measurements were done in two unstressed and stressed condition using standardized net handling as the stressor. A significant positive linear correlation between cortisol levels and eGFP mRNA levels was observed (r> 0.9). Based on eGFP mRNA levels in unstressed and stressed larvae two predictive models were trained (Random Forest and Logistic Regression). Both these models could correctly predict the blunted stress response upon exposure to BPA, VIN, FLX and the negative control, DEX. The negative predictive value (NPV) of these models were 100%. Similar NPV was observed when the predictive models trained based on the mRNA levels of the eleven assessed genes. Measurement of whole-body fluorescence intensity signal was not significant to detect blunted stress response. Our findings support the use of SR4G transgenic larvae as an *in vivo* biomonitoring model to screen chemicals for their stress-disrupting potentials. This is important because there is increasing evidence that brief exposures to environmental pollutants modify the stress response and critical coping behaviors for several generations.

## Introduction

The eggs and embryos of aquatic vertebrates such as fish and frogs are directly exposed to the potential adverse effects of environmental contaminants because of their external fertilization and development (1). Many potential environmental hazards originate from municipal and industrial waste management facilities because both treated and untreated effluents may be released to rivers and other water reservoirs (2, 3). Amongst the most studied environmental contaminants endocrine disrupting compounds (EDCs), pharmaceuticals (PCs) and agricultural pesticides have shown to affect many aspects of embryonic development in aquatic vertebrates (4). The EDCs with their potential to interfere with hormonal systems are of high risk, particularly to a developing embryo (5–8). These EDCs not only affect the survivability and normal development of an exposed embryo, but there is an increasing body of evidence that support long-lasting effects of such an exposure to the adulthood and even to the descendant generations (5, 7, 9, 10). For example, reduced fertility, altered sexual behaviour, altered locomotion, and reduced stress and anxiety-like responses have been reported in adult teleost exposed to EDCs during early life stages (11, 12).

Zebrafish embryos and larvae are considered as valuable animal models in toxicological assessments, particularly for their potential to bridge *in vitro* and *in vivo* studies (1, 13). Zebrafish embryos rapidly develop from a single-cell stage to a fully functional multi-organs larvae in 4 to 5 days; therefore, could be an excellent model to study developmental toxicology (14). Their external development makes many experimental manipulations more feasible compared to mammalian models (14). Moreover, high fecundity provides adequate sample size for high-throughput studies which is less convenient with many other *in vivo* animal models (14). The traditional assessment of morphological changes upon exposure to different arrays of potential toxicants is well-documented for zebrafish embryos, and automation protocols are described (15). Overall, the small size, easy dosing, moderate skin transparency, and rapid development, further contribute to the choice of zebrafish for high-throughput screening for potential toxicants (13, 16, 17).

Gene expression analysis, behavioural monitoring, and fluorescence imaging of inducible transgenes are amongst recently established endpoints for biomonitoring approaches using zebrafish (13). Based on the Organization for Economic Co-operation and Development (OECD) guidelines several members of the teleost family including zebrafish (*Danio rerio*), fathead minnow (*Pimephales promelas*), Japanese medaka (*Oryzias latipes*), bluegill (*Lepomis macrochirus*), and rainbow trout (*Oncorhynchus mykiss*) are used in Acute Fish Toxicity test as a part of “hazard and environmental risk assessment” (18). Recently, a modified version of this test using zebrafish embryos (zebrafish embryo acute toxicity test, ZFET), has been validated by regulatory bodies for environmental exposure risk assessment; given its high predictive capacity (4, 18).

Recently, Krug *et al.* created a stress-inducible transgenic zebrafish line (SR4G; Stress receptor 4hr enhanced eGFP), which expresses an enhanced version of green fluorescent protein (eGFP) under the control of the CMLC (cardiac myosin light chain 2) promoter and six consecutive glucocorticoid response elements (GREs) (19). The short 4 hours half-life of the expressed eGFP allows one to capture the dynamics of stress response under different exposure modes (19). The whole-body ubiquitous expression of eGFP provides high levels of fluorescence intensity which is detectable by standard laboratory imaging equipment. The SR4G transgenic line has been used as a model to study neuropsychiatric diseases, including major depressive disorder, generalized anxiety disorder and substance use disorder (19–22).

The response to stressful stimuli consists of a short acting response (driven by the sympathetic adrenomedullary system) and a secondary response regulated by hypothalamic-pituitary-adrenal system *via* corticosteroid production and action (23). The later is the focus of our study. We tested the hypothesis of using whole-body eGFP mRNA and the whole-body eGFP fluorescence in the SR4G transgenic line as alternatives for the whole-body cortisol measurement to assess stress disruption. As a proof-of-concept, two EDCs with known stress-disrupting properties, bisphenol A (BPA), a common plasticizer; and vinclozolin (VIN), a common fungicide (10, 24–28) were selected. Fluoxetine (FLX) was also studied because is it a widely used pharmaceutical with high stress-disrupting potential (29–32). The glucocorticoid receptor (GR) agonist (hydrocortisone; CORT) was used as positive control for the stress response. To suppress endogenous cortisol production dexamethasone (DEX) was used. This group was considered as the negative control. Moreover, eleven genes belonging to the immediate early response class of genes (IEGs) and stress-axis were selected as the second alternative for detection of stress disruption. The whole-body levels of cortisol, eGFP fluorescence, levels of mRNA for eGFP mRNA and eleven target genes were studied. All measurements were performed before (unstressed) and after a standardized handling stressor (stressed). Our findings indicate that models based on the whole-body eGFP mRNA can predict stress disruption in zebrafish larvae and may be valuable tools for medium- to high-throughput screening in tier-1 risk assessment studies.

## Materials and Methods

### Animals and Husbandry

The SR4G transgenic zebrafish line was kindly provided by Dr. Karl Clark (Mayo Clinic, Minnesota, USA) and Dr. Xiao-Yan Wen (University of Toronto, Ontario, Canada). The one-day post-fertilization embryos were transferred to Ottawa and placed in the quarantine room of the aquatic facility following regulations of the Canadian Council on Animal Care and approved by the Animal Care and Veterinary Services (ACVS) of the University of Ottawa. The embryos hatched and were kept in the quarantine facility until they reached six months of age. These zebrafish were used to establish our founder generation. The embryos and adults were kept in controlled temperature (28°C), and a 14:10 hour light/dark cycle and fed 2-3 times daily.

### Chemicals and Exposure Concentrations

All chemicals were purchased from the commercial sources (Millipore-Sigma, Burlington, Massachusetts, USA). The exposure doses were determined based on several pilot studies using the published LC10 and/or therapeutic doses of the specific compounds. The final exposure concentration for BPA, and VIN were at environmentally relevant concentrations based on the previously published literature (22, 33–38). The working concentration of dexamethasone (DEX) was determined based on the preliminary studies performed in our lab. Several different dilutions were prepared, and the survival rate pre- and post handling stress assessed in 7-dpf zebrafish larvae determined. The cortisol levels were measured using ELISA (described in section 2.4). The lowest effective concentration of dexamethasone that could result in suppression of cortisol production was thus determined to be 0.2 nM. The FLX concentration chosen was based on the clinically relevant dose used in a previous study in our lab (31), which was within the lower range found in the cord blood of pregnant women receiving FLX (39–41). The SR4G transgenic zebrafish embryos allowed to reach six days post-fertilization with daily fresh embryo medium change. On the evening of the 6^th^ day, embryos were treated with either of the following compounds and the assigned concentrations for a total of 18 hours (overnight) of exposure: BPA, 5 µM; VIN, 17 µM; DEX, 0.2 nM; cortisol (CORT), 10 µM; and FLX, 54 µg/L. The control groups only received the final concentration of 0.001% of DMSO as the vehicle for BPA, VIN and DEX, and the final concentration of 0.005% of ethanol as the vehicle for the FLX group. To mimic our previous studies (Vera-Chang et al), another FLX-exposed group (FLX-6D) was generated by exposing 0-day post fertilization (dpf) embryos to 54 µg/L of fluoxetine for six days (6 dpf) with daily change of embryo medium containing the freshly prepared FLX from the stock solution. The associated control group (Ethanol-6D) only received the vehicle with the same daily embryo medium changes. The exposed treatment and control groups were further divided into four subgroups, unstressed (0 minutes), stressed for cortisol study (30 minutes post handling stress), stressed for mRNA study (60 minutes post handling stress) and stressed for fluorescent study (120 minutes post handling stress). The tank of each group was prepared on the day of embryo collection and kept in separate shelves in the incubator to avoid unwanted stress in the unstressed groups. The unhatched or dead embryos/larvae were collected once daily.

### Stress Conditions and Sample Collection

Following the 18hr overnight exposure, the unstressed groups were immediately euthanized in ice-cold water; and samples were collected for cortisol measurement, qPCR, and fluorescence measurements (22-28 larvae in each tube; n=6). After removal of the excess medium from each tube, samples were immediately placed on dry ice and later transferred to -80°C freezer until analysis. The stressed groups underwent a modified net-handling procedure previously described (42). Briefly, the larvae from each group were exposed to air for 1 minute trapped in a mesh net, afterwards returned to the embryo medium for 3 minutes of rest, following by another 1-minute exposure to air. The time points for sample collections were determined based on pilot studies performed in our lab. The collection times post-handling stress varied according to the specific endpoint being measured: cortisol, 30 minutes; for mRNA, 60 minutes; and for eGFP fluorescence,120 minutes. The excess water was removed, and the collected samples were kept in -80 °C until later analysis.

### Whole-Body Cortisol Measurement

Whole-body cortisol was measured using the Cayman Cortisol ELISA kit (Cat# 500360 Cayman Chemicals, Michigan, USA) based on the manufacturer’s recommendations. Cortisol was extracted from pools of 22-28 larvae by an ether lipid extraction protocol described elsewhere (43). Briefly, about 200 ul of freshly prepared PBS was added to each sample and samples were sonicated (Qsonica- q700, CT, USA) at Hz for two periods of 10 s while incubating on ice. Afterwards, ether was added about three times the initial volume (600 ul), samples were centrifuged on 4°C at 4000 rpm for 5 minutes, and supernatants were collected. This procedure was repeated three times for each sample. The total collected supernatants were placed under the chemical hood for ether evaporation overnight. Following full evaporation, 50 µl of ELISA buffer was added to each sample and incubated on 4°C for 24-30 hours with occasional vortex for full resuspension of the lipid extracts. The resuspended samples were used for cortisol measurement. Samples with a high amount of cortisol which fell in the non-linear part of the standard curve, where diluted and measured again. Based on the spiked samples with known amounts of cortisol (from standard solutions provided in the ELISA kit) the extraction efficiency was determined as >85%.

### Total RNA Extraction and Quantitative PCR

Total RNA extraction from all samples was carried out using Qiagen Rneasy mini kit, (Qiagen, Hilden, Germany) based on the manufacturer recommendations. The extracted total RNA samples were kept in -80 °C freezer until further experimentations. A fraction of total RNA extracts for each sample was converted to cDNA using Qiagen QuantiNova Reverse Transcription Kit (Qiagen, Hilden, Germany), based on the manufacturer recommendations. The cDNA samples were kept in -80 °C freezer until further experimentations. Eleven genes related to stress-axis and IEGs were studied; including: *crhb, bdnf, egr2a, fkbp5, fosab, fosl1a, htr1b, npas4a, nr4a1, per2, and rorcb.* For qPCR experiments, forward and reverse primers for the 11 selected genes were designed using the NCBI Primer-BLAST tool (https://www.ncbi.nlm.nih.gov/tools/primer-blast/). The sequence of each of the primers is shown in **Supplementary Table 2**. The forward and reverse primers sequence for eGFP and *eef1a1l1* (reference or housekeeping gene) were adopted from the previously published data (19). All oligonucleotides were synthesized by Integrated DNA Technologies (Coralville, Iowa, USA) (**Supplementary Table 2**). The qPCR experiments were conducted according to SYBR green chemistry principle using the SsoAdvanced universal SYBR Green Supermix and Bio-Rad CFX Thermal Cycler (Bio-rad, Hercules, California, USA) according to the manufacturer’s recommendations. The efficiencies for qPCR runs were between 98-103%, with a slope between -3.58 to -3.10. Experiments were conducted using the same batch of materials in a single day. All experiments performed with N=6 replicates

### Whole-Body Fluorescence Measurement

Freshly prepared PBS (200 µl) containing a protease inhibitor cocktail mix (1:1000 dilution of #P8340-5ML, Millipore-Sigma, Burlington, Massachusetts, USA) was added to all larval samples before sonication. Samples were sonicated (Qsonica- q700, CT, USA) at 20 kHz for two periods of 10 seconds while incubated on ice. The samples were centrifuged afterwards at 10000 rpm on a refrigerated centrifuge at 4°C for 4 minutes. Fifty microliters of the supernatant were transferred into a well of a 96 well flat bottom plate, and fluorescence signal of eGFP was measured by 485/20 filter for excitation and 530/25 filter for emission using Cytation 3 multi-well reader (# BTCYT3V, Biotek, Winooski, Vermont, USA).

### Whole-Body Fluorescence Imaging

The 4dpf zebrafish larvae were subjected to the handling stress routine. The unstressed (0 minutes) and stressed larvae were anaesthetized using Tricaine** **mesylate as described in the ZFIN database (https://wiki.zfin.org/display/prot/TRICAINE) and fixed in a six-well plate containing low melting point agarose (44). The heartbeat of the larvae was monitored throughout the experiment to assure stable anaesthesia. The fluorescence imaging was performed using 485/20 filter for excitation and 530/25 filter for emission using Cytation 3 multi-well reader (Biotek, Winooski, Vermont, USA). Serial images were acquired every 15 minutes. The fluorescence intensity unit was measured on a single representative image using ImageJ software (version 1.8_172; NIH; https://imagej.nih.gov/ij/) at four different anatomical regions. The areas of interest were encompassing the telencephalon, diencephalon, right olfactory bulb, and left olfactory bulb. To measure similar regions, all images were scaled to a similar pixel ratio using the SCALE function of ImageJ. The measuring areas were drawn on the highest fluorescence intensity region using the image acquired at 60 minutes past the handling stress. The drawn areas were saved using the ROI (region of interest) function of ImageJ. The same ROI setting was used for all other images. In each anatomical section, ten similarly sized areas were drawn and measured. The mean of fluorescence intensity (mean grey value) and the standard deviation for each anatomical section was calculated. The statistical analysis performed as described below.

### Statistical Analysis

All statistical analysis was performed by XLSTAT (v.2020.2, Addinsoft Inc, N.Y. USA). To determine if datasets are normally distributed the Shapiro-Wilk or Jarque-Bera test were performed. Also, the homogeneity of variances was assessed using Levene’s test. Given the design of each experiment, one-way ANOVA or two-way ANOVA was as required. All tests were performed with six replicates to determine the level of significance followed by Tukey’s *post-hoc* test for pairwise comparisons. The level of significance was selected as p ≤ 0.05.

The Pearson test was performed to assess linear correlation of cortisol levels and eGFP mRNA levels. For predictive modeling, the statistical modelling was performed by Binomial Logistic Regression (LOG) and regression Random Forest (RDF). The R^2^ and Out-Of-Bag (OOB) ratio were used to assess the fitness of each model to the original data set and the predicted data set for LOG and RDF, respectively.

## Results

### eGFP Signals as Surrogates for Cortisol Could Detect the Stress Response in SR4G Zebrafish Larvae

**Figure 1:**
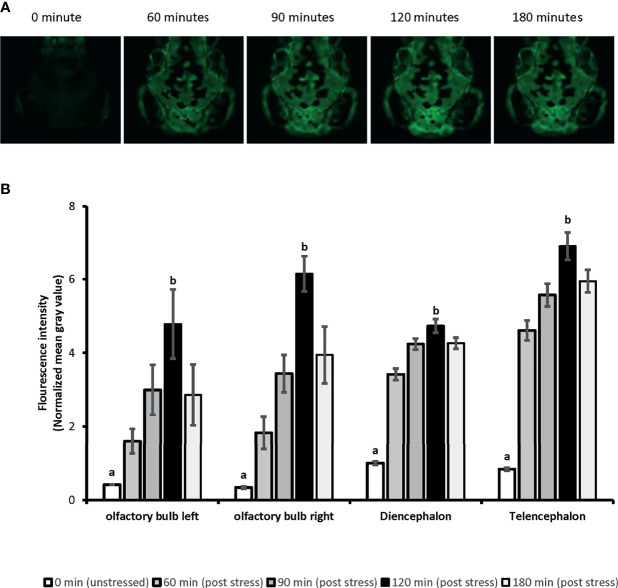
The time-lapse fluorescence imaging captures the ubiquitous eGFP expression in 4dpf SR4G transgenic zebrafish line following the handling stress. Panel **(A)** shows the direct fluorescent imaging of 4dpf SR4G transgenic larva head at five different time-points after the handling stress. Panel **(B)** shows the quantified fluorescence intensity (mean grey value) after the handling stress in four different anatomical regions; diencephalon, telencephalon, left olfactory bulb and right olfactory bulb at five different time-points. Means (± SEM) marked with different letters (a, b) are significantly different within each anatomical position; *p* < 0.01; N=10.

Whole-body cortisol and eGFP mRNA levels were measured to determine the appropriate time for sample collection. Our analysis showed that the whole-body cortisol levels increased up to 4.2-fold, 30 minutes after the handling stress compared to the related unstressed condition in both vehicles used (Ethanol and DMSO), respectively [F(1,20)=34.69, p=9^E0-6^]. Similar significant increase in the whole-body eGFP mRNA levels were also observed. A 4.4-fold and 3-fold increase in eGFP mRNA levels observed 60 minutes after the handling stress in both vehicles used (Ethanol and DMSO); respectively [F(1,20)=25.32, p= 6.8^E0-5^]. Further analysis showed that the cortisol levels returned to pre-stress (unstressed) levels by 90 minutes. A similar return to pre-stress (unstressed) levels was observed for eGFP mRNA by 120 minutes post handling stressor. The imaging analysis on 4-dpf larvae head showed increased eGFP fluorescence intensity in a timely manner for 120 minutes (**Figure 1**). The mean of fluorescence intensity was consistently elevated until 120 minutes post-handling stress across all four anatomical regions when compared to the control (unstressed state): left olfactory bulb [5-fold], right olfactory bulb [6-fold], diencephalon [5-fold] and telencephalon [6-fold]. It was determined that collecting samples at 120 minutes post handling stressor could significantly capture the increased eGFP fluorescence signal in the four anatomical region of the larvae brain [Telencephalon: F(4,45)=40.97, p=1.86^E0-14;^ Diencephalon: F(4,45)=41.03, p=1.86^E0-14^; left olfactory bulb: F(4,45)=62.93, p=4.11^E0-18^; right olfactory bulb: F(4,45)=60.5, p=1.56^E0-17^] (**Figure 1**).

**Figure 2:**
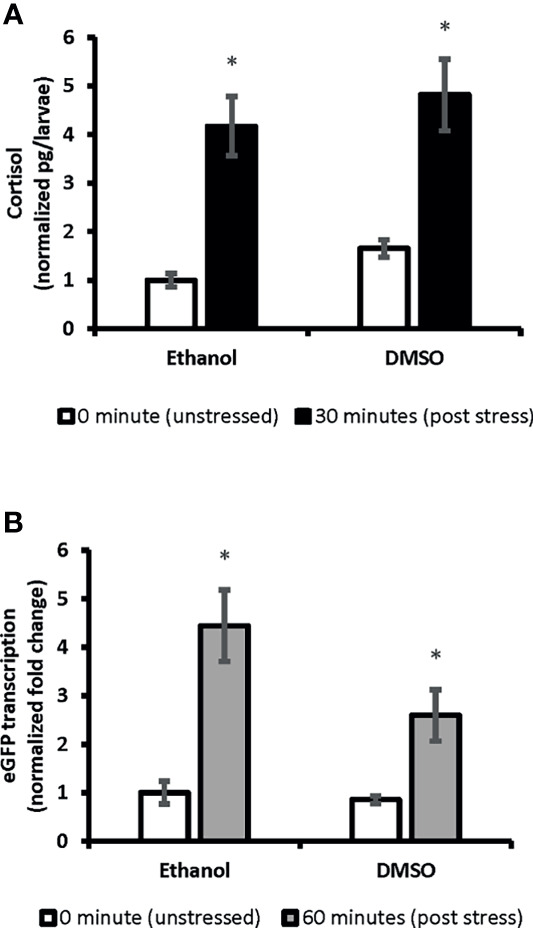
The correlation between whole-body cortisol and total eGFP mRNA levels in SR4G transgenic zebrafish larvae. Panel **(A)** shows normalized whole-body cortisol levels and panel **(B)** shows normalized whole-body eGFP mRNA levels in the two control groups, Ethanol and DMSO, under the two unstressed and stressed conditions. Two-way ANOVA was performed to evaluate the effect of treatments and stress conditions on whole-body cortisol levels and whole-body eGFP mRNA levels. Means (± SEM) marked by an asterisk (*) are significantly different within each category (whole-body cortisol or whole-body eGFP mRNA study); *p ≤* 0.0001; N=6. Ethanol: overnight exposure to 0.005% ethanol; DMSO: overnight exposure to 0.001% DMSO. The time points,30 min or 60 min, show the time-lapsed following the handling stress before euthanizing the subjects for sample collection. Each sample contained a pool of 22-28 of 7dpf SR4G transgenic zebrafish larvae. The correlation coefficient between whole-body cortisol and whole-body eGFP mRNA determined by the Pearson test and a positive correlation with r=0.947 achieved.

We showed ubiquitous expression of eGFP in SR4G larvae. Our findings showed that eGFP expression responded promptly to the handling stressor both in transcription level (mRNA) and translation level (protein fluorescence signal). Two different time points (60 minutes for mRNA and 120 minutes for fluorescence) were determined to capture the significant elevation of eGFP signal which were comparable to elevation of cortisol amounts. A significant positive correlation was observed between cortisol and eGFP mRNA levels in SR4G larvae (r=0.947, p=2.53^E0-12^). A similar significant positive correlation was also observed between cortisol and eGFP fluorescence intensity in telencephalon (r=0.826, p<0.001) and diencephalon (r=0.895, p<0.001) of SR4G zebrafish larvae. Our findings showed that it is possible to use eGFP signals (mRNA and fluorescence intensity unit) as alternative routes to cortisol measurement following a handling stressor in SR4G zebrafish larvae (**Figure 2**).

### Whole-Body Cortisol and eGFP mRNA Responses to the Handling Stress Are Blunted Following Exposure to BPA, VIN, FLX and DEX

**Figure 3:**
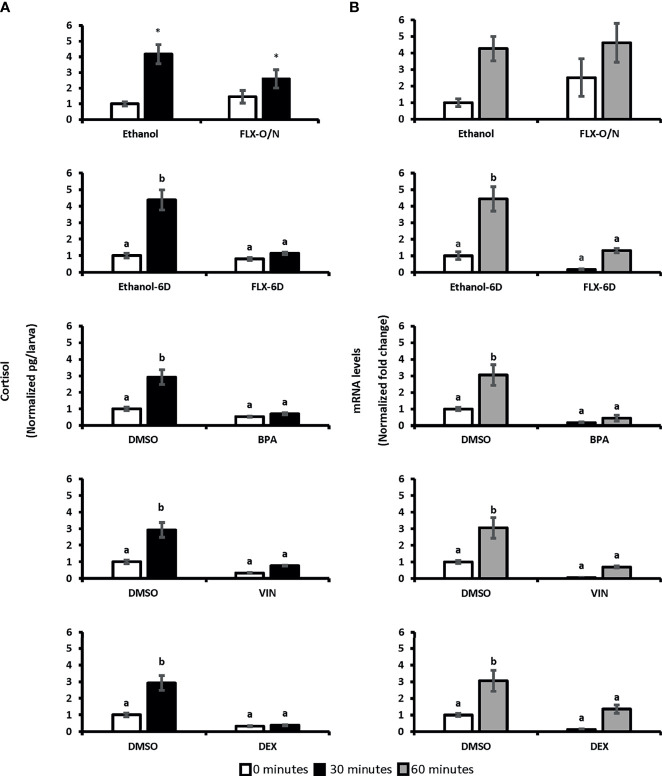
Whole-body cortisol and eGFP mRNA levels in SR4G larvae exposed to different chemicals before and after handling stress. Panel **(A)** shows the whole-body cortisol (normalized pg/larvae) measured before and after the handling stress following exposure to different chemicals. The white bars represent cortisol levels in the unstressed condition (sacrificing subjects at 0 minutes before the handling stress) and the black bars represent cortisol levels 30 minutes after the handling stress. Panel **(B)** shows the eGFP mRNA levels (normalized fold change) measured before and after the handling stress following exposure to different chemicals. The white bars represent eGFP mRNA levels in the unstressed condition (sacrificing subjects at 0 minutes before the handling stress) and the gray bars represent eGFP mRNA levels 60 minutes after the handling stress. Two-way ANOVA was performed between each treatment group and its related control group in both unstressed and stressed state. No statistical comparison was performed between different treatment groups. Tukey’s *post-hoc* test followed the significant ANOVA results for pairwise comparisons. Means (± SEM) with different letters (a, b) are significantly different; p ≤ 0.05; N=6. Each sample contained a pool of 22-28 7dpf SR4G transgenic zebrafish larvae. Ethanol was used as the vehicle for FLX with a final concentration of 0.005%. DMSO was used as the vehicle for BPA, VIN, and DEX with a final concentration of 0.001%. The DMSO data in each graph is the same data portrayed for comparison purposes. FLX-O/N, overnight exposure to fluoxetine; FLX-6D, daily exposure to fluoxetine from 0 to 6 days post fertilization(dpf); Ethanol-O/N, overnight exposure to ethanol; Ethanol-6D, daily exposure to ethanol from 0dpf to 6dpf; BPA, overnight exposure to bisphenol A; VIN, overnight exposure to vinclozolin; DEX, overnight exposure to dexamethasone; DMSO, overnight exposure to DMSO. *Significantly different (p<0.05) compared to unstressed state but not significant in 2-way ANOVA.

Two-way ANOVA was conducted on the influence of exposure to different chemicals with potential stress-altering properties (BPA, VIN, DEX, and FLX) on the cortisol levels in 0 minutes (unstressed) and 30 minutes following the handling stress (stressed). A significant difference in whole-body cortisol levels following handling stress observed forBPA [F(1,20)=27.79, p=3.69^E0-5^], VIN [F(1:20)= 31.21, p=1.8^E0-5^], DEX [F(1:20)= 40.06, p=3.5^E0-6^], and FLX-6D [F(1,20)=21.67, p=0.0001] when compared to the vehicles (DMSO or ethanol). Such significant difference was not observed after overnight exposure to FLX [FLX-O/N; F(1,20)=1.19, p=0.28] Pairwise comparison showed that cortisol levels 30 minutes after the handling stress increased up tp 4.2-fold in vehicle groups (p ≤ 0.01). Such significant increase was not observed for BPA (0.7-fold, p>0.05), VIN (0.8-fold, p>0.05), FLX-6D (1.85, p>0.05), and DEX (0.4-fold, p>0.05). Our findings showed that the exposure to DEX, BPA, and VIN resulted in a blunted cortisol response in SR4G zebrafish larvae following the handling stress. A similar blunted cortisol levels was observed for FLXonly after a prolonged exposure (6 days) and not short overnight exposure (**Figure 3**).

To determine if eGFP mRNA follows a similar pattern as cortisol, two-way ANOVA was conducted on the influence of exposure to BPA, VIN, FLX, and DEX on the eGFP mRNA levels in 0 minutes (unstressed) and 60 minutes (stressed) following the handling stress. A significant difference was observed for BPA [F(1,20)=22.63, p=0.0001], VIN [F(1:20)= 16.41, p=0.0001], DEX [F(1:20)= 11.86, p=0.002], and FLX-6D [F(1,20)=21.24, p=0.0001] compared to vehicle groups. Such significant effects on whole-body eGFP levels was not observed following overnight exposure to FLX [FLX-O/N: F(1,20)=0.7, p=0.40]. Whole-body eGFP mRNA levels 60 minutes after the handling stress increased up to 4.6-fold in the vehicle groups (p ≤ 0.01). Such significant increase was not observed for BPA, VIN, FLX-6D, and DEX. An increase of 4.6-fold was observed in the FLX-O/N sixty minutes after the handling stress which was similar to a 4.3-fold increase in the ethanol group in the same time point(p≥0.05). Our findings showed that eGFP mRNA levels follow a similar pattern of whole-body cortisol levels upon exposure to BPA, VIN, FLX-6D, and DEX (**Figure 3**).

To evaluate the response of SR4G larvae to glucocorticoid agonists, whole-body cortisol and eGFP mRNA were measured following overnight exposure to hydrocortisone (CORT). As expected, the levels of cortisol and eGFP mRNA were high in pre-and post stress conditions (**Supplementary Figure 1**). These findings once more showed the close correlation between whole-body cortisol and whole-body eGFP mRNA levels.

### Whole-Body eGFP mRNA Levels Are Predictive of the Altered Stress Response in SR4G Larvae

The whole-body eGFP mRNA levels in stressed and unstressed conditions in ethanol group were used as the training data set to create two predictive models based on random forest (RDF) and logistic regression (LOG). The stressed condition was used as the qualitative dependent variable. Based on the whole-body cortisol levels measured upon exposure to different chemicals (FLX-6D, FLX-O/N, BPA, VIN, DEX, and CORT) the significant stress response (higher cortisol levels compared to the unstressed state, p ≤0.05) was assigned a binary value 1 (one), and the statistically non-significant measures were assigned a binary value 0 (zero). The out-of-bag (OOB) error estimate for RDF was 0.4, and the validation misclassification rate was 0.00. The R² for LOG was 0.774. The positive predictive value (PPV) and the negative predictive value (NPV) was calculated for both models for each treatment group (**Table 1**). Our findings showed that the models based on whole-body eGFP mRNA levels could predict the altered stress response upon exposure to different chemicals.

**Table 1:** Prediction of stress response based on the whole-body eGFP mRNA levels using regression random forest (RDF) and logistic regression (LOG) model trained by ethanol (control) group.

Ethanol (control)			Predicted Stress Status																	
			FLX_O/N			FLX-6D			DMSO			BPA			VIN			DEX		
Samples	FC	Stress	FC	LOG	RDF	FC	LOG	RDF	FC	LOG	RDF	FC	LOG	RDF	FC	LOG	RDF	FC	LOG	RDF
Uns-1	2.05	0	9.33	1	1	0.23	**0**	**0**	1.33	**0**	**0**	0.16	**0**	**0**	0.10	**0**	**0**	0.06	**0**	**0**
Uns-2	0.90	0	5.69	1	1	0.16	**0**	**0**	0.72	**0**	**0**	0.24	**0**	**0**	0.08	**0**	**0**	0.36	**0**	**0**
Uns-3	0.64	0	1.27	**0**	**0**	0.39	**0**	**0**	0.93	**0**	**0**	0.37	**0**	**0**	0.04	**0**	**0**	0.10	**0**	**0**
Uns-4	0.27	0	0.74	**0**	**0**	0.10	**0**	**0**	1.09	**0**	**0**	0.07	**0**	**0**	0.06	**0**	**0**	0.04	**0**	**0**
Uns-5	0.72	0	1.12	**0**	**0**	0.24	**0**	**0**	0.80	**0**	**0**	0.16	**0**	**0**	0.00	**0**	**0**	0.07	**0**	**0**
Uns-6	1.41	0	0.04	**0**	**0**	0.07	**0**	**0**	1.28	**0**	**0**	0.09	**0**	**0**	0.01	**0**	**0**	0.17	**0**	**0**
St-1	6.95	1	12.81	**1**	**1**	0.99	**0**	**0**	3.93	**1**	**1**	0.00	**0**	**0**	0.55	**0**	**0**	0.06	**0**	**0**
St-2	4.18	1	5.80	**1**	**1**	1.32	**0**	**0**	0.84	0	0	0.00	**0**	**0**	0.58	**0**	**0**	0.36	**0**	**0**
St-3	1.57	1	2.77	**1**	0	1.64	**0**	**0**	1.24	0	0	1.01	**0**	**0**	0.86	**0**	**0**	0.10	**0**	**0**
St-4	2.89	1	2.80	**1**	0	1.55	**0**	**0**	5.00	**1**	**1**	0.79	**0**	**0**	0.78	**0**	**0**	0.04	**0**	**0**
St-5	5.32	1	3.60	**1**	**1**	2.32	1	**0**	4.45	**1**	**1**	0.01	**0**	**0**	0.57	**0**	**0**	0.07	**0**	**0**
St-6	5.74	1	5.60	**1**	**1**	1.65	**0**	**0**	3.30	**1**	**1**	0.94	**0**	**0**	0.92	**0**	**0**	0.17	**0**	**0**
**PPV**				**75%**	**67%**		**N/A**	**N/A**		**100%**	**100%**		**N/A**	**N/A**		**N/A**	**N/A**		**N/A**	**N/A**
**NPV**				**100%**	**67%**		**100%**	**100%**		**75%**	**75%**		**100%**	**100%**		**100%**	**100%**		**100%**	**100%**

FC, fold change; FLX-6D, daily exposure to from 0 to 6 days post fertilization(dpf); FLX-O/N, BPA, VIN, DEX, CORT representing overnight exposure to fluoxetine, bis-phenol A, vinclozolin, dexamethasone, and cortisol, respectively. LOG, logistic regression, RFD, random forest. PPV, positive predictive value; NPV, negative predictive value. Uns, unstressed sample; St, stressed sample. (LOG; R^2^, 0.774) (RDF; OOB, 0.4).

N/A, not applicable, the PPV can not be calculated.

The correct predications (true) are shown by bold values (1 or 0).

### Whole-Body mRNA Levels of Genes Related to Stress, Depression and Neurogenesis Are Predictive of Altered Stress Responses Following Chemical Exposures in SR4G Transgenic Zebrafish Larvae

The potential use of a panel of pre-selected genes to predict the stress response was also evaluated. Eleven genes for which their human orthologues were associated to stress response, development of depression, and neurogenesis were used. The stressed condition was used as the qualitative dependent variable. The significant stressed condition (based on whole-body cortisol amounts) was assigned binary value 1 (one), and the nonsignificant measure was assigned a binary value 0 (zero). The whole-body mRNA levels of the selected genes in ethanol group in both stressed and unstressed conditions were used to train the predictive model. Two models based on RDF and LOG were created. The out-of-bag (OOB) error estimate for RDF was 0.4, and the validation misclassification rate was 0.00. The R² for LOG was 0.741. The mRNA levels of selected genes in both unstressed and stressed conditions were imported to the model regardless of their level of significance. our findings showed that both predictive models could determine the altered stress response upon exposure to different chemicals (FLX, BPA, VIN, DEX, and CORT) (**Supplementary Table 2**). We showed that although exposure to BPA, VIN, FLX (only the prolonged exposure), and DEX resulted in significant blunted cortisol response (**Figure 3**), not all selected genes were significantly affected (**Supplementary Figures 2**, **3**).

### Whole-Body Fluorescence Measurements Do Not Effectively Detect Disruption of the Stress Response Following Chemical Exposure

The fluorescence intensity was measured in the crude protein extracts in pools of 7dpf SR4G transgenic larvae at 0 minutes (unstressed), and 120 minutes after handling stress. The two-way ANOVA did not show any significant effects for both variables (stress condition and chemical exposure). Our findings indicated that fluorescence intensity measured in the proposed way was not sensitive and could not adequately discriminate between stressed and unstressed states, SR4G zebrafish larvae (**Supplementary Figure 3**).

## Discussion

Our findings revealed that acute exposure to several environmental contaminants and pharmaceuticals can disrupt normal activity of the cortisol-mediated stress response in zebrafish. There is increasing evidence that SSRIs and other EDCs may disrupt the normal functioning of the endocrine stress axis (5, 10). In some cases, this may trans-generationally inherited, perturbing coping behaviours and physiological control mechanism for generations (27, 31). The stress response is thus critical as it is the driving force for organismal adaptation and survival (45). The development of diverse psychological and behavioral phenotypes is strongly associated with disruption of the stress response (46, 47). Development of robust methods to monitor stress-disrupting chemicals is thus of paramount importance. For this purpose, we have evaluated the SRG4 zebrafish transgenic line and have developed a strategy for screening EDCs.

Commonly used immunoassays may have cross-reactivity with other steroids and suffer from inter-assay and intra-laboratory variability (48, 49). Other endpoints have been suggested for evaluation of the stress response such as behavioural (e.g., explosive activity, locomotor activity), physiological (e.g., heart rate, blood pressure, pain), metabolic (e.g., glucose, insulin, ketone bodies), neurochemical (e.g., catecholamines, serotonin, dopamine), and endocrine (e.g. ACTH, CRH, vasopressin) (50). However, the challenges of measuring these endpoints may be equal to or greater than immunoassays. Alternatively, the transcriptional analysis of stress-axis genes has been suggested (50). Another alternative is behavioural analysis, however, strict study design is necessary to achieve replicable results (51).

We suggest that measuring eGFP mRNA in SR4G zebrafish model is a good alternative assessment strategy for stress and its disruption and incorporates several aspects of other. assays, including the *in vitro/in vivo* bridge. The two predictive models, random forest (RDF) and logistic regression (LOG), trained using whole-body eGFP mRNA in a control group (ethanol), could correctly predict the blunted stress response upon exposure to BPA and VIN in 7-dpf zebrafish larvae. The overnight exposure to BPA and VIN blunted the increase of whole-body cortisol and eGFP mRNA after a standardized handling stressor compared to the control groups. The negative predictive value of 100% showed that the proposed models could effectively identify these stress-disruptive compounds.

The generated predictive models could also discriminate between the two FLX exposure scenarios. A common antidepressant, FLX, elicits both its therapeutic and adverse effects after prolonged exposure (52). The overnight (18 hours) exposure to FLX did not significantly affect whole-body cortisol levels and, consequently, whole-body eGFP mRNA levels. On the other hand, prolonged developmental exposure (0 to 6-dpf in zebrafish) to FLX resulted in a blunted stress response; evident by a significant decrease in the whole-body cortisol and the whole-body eGFP mRNA after a handling stress in zebrafish larvae. This confirmed previous research demonstrating that 6 days exposure to FLX blunted the stress response, an effect that can persist across generations in zebrafish (31).

It was found that the predictive models (RFD and LOG) based on mRNA levels of 11 different genes had similar NPV to the models based on eGFP mRNA alone. The selected genes are associated with stress responses, development of depression, and neurogenesis in mammalian and teleost models (53–55). Several of the selected transcripts including *fosab, egr2a, nr4a1, and npas4a* are considered members of the IEG class. A variety of external stimuli could induce the expression of IEGs, which is rapid, transient, and protein synthesis-independent (56). The dysregulation of these genes in human and rodent models is associated with altered stress responses and impaired neural plasticity (53, 56–60). Our study showed that the disruption of the normal stress response upon overnight exposure to BPA and VIN resulted in a marked downregulation of *fosab* after handling stress. Downregulation of *fosab* was observed after the developmental exposure to FLX (FLX-6D group). Similar downregulation was observed in the DEX group that had suppressed cortisol production. Upregulation of *Fos* (orthologue of *fosab*) has been observed in specific brain regions following a seizure, tactile sensory stimulation, visual stimulation, cocaine administration, and nursing newborn offspring in rodent models (60, 61). Alternate splicing and downregulation of Fos have been reported following chronic stress and FLX therapy in rodent models (62).

Several of the target genes we assessed were downregulated following overnight exposure to BPA, VIN, and DEX in unstressed larvae, including *per2*, *egra2* and *fosl1*. Similar downregulation in unstressed condition was observed following the 6-day developmental exposure to FLX, and interestingly not the overnight exposure to FLX. Our findings showed a distinct downregulation of *npas4, nr4a1, per2*, and *rorcb* following developmental exposure to FLX and overnight exposure to VIN in both stressed and unstressed larvae. These four genes are involved in neurogenesis and neural activity in rodent models (60, 63). This pattern was not observed for BPA and DEX, demonstrating some specificity of the responses, which might be explained by differences in the mechanism of action of these compounds.

We also report that *bdnf, crhb*, and *htr1b* were not affected by exposure to the studied compounds. In mammals, brain-derived neurotrophic factor (BDNF) is a critical gene in regulating neural growth, synaptic maturation, and neurogenesis (64). Although BDNF is not precisely an IEG member, it has activity-dependent expression and responds to a wide range of external and internal stimuli (63, 65). Decreased levels of BDNF are associated with the development of major depressive disorder, and its increase following antidepressant therapy is measured as a response to therapy criteria in humans (66). The induction of neurogenesis resulting from TrkB (BDNF receptor) activation is implicated in memory formation, cocaine addiction behaviours, and recovery after stroke in mammalian models and human studies (63). We observed a wide variation in *bdnf* mRNA levels across samples and treatments in our study. The only significant upregulation of *bdnf* was observed upon exposure to VIN when the unstressed conditions were compared. This could be either due to measuring a gene transcription in the whole-body settings (67) or a different response time for *bdnf* in zebrafish compared to other models. In our study, *crhb* and *htr1b* were not affected by the treatments. However, downregulation of *htr1b* following developmental exposure to FLX was observed when the comparison was performed only in an unstressed condition. There is a discrepancy of findings regarding *htr1b* alteration following exposure to SSRIs, with some studies showing an apparent dysregulation while others do not report such changes (68, 69). The exact reason for such discrepancies is presently unknown. The study of mRNA level of the selected 11 genes had similar predictive value as the study of eGFP mRNA only in SR4G transgenic larvae.

We also measured the whole-body fluorescence signal in the crude homogenized pooled sample of SR4G transgenic larvae. Our experiments using microscopic fluorescence imaging showed that measurement of fluorescence intensity in four anatomical regions (including telencephalon and diencephalon) could discriminate between the stressed and unstressed conditions and had a positive correlation with whole-body cortisol levels in SR4G zebrafish larvae. However, using the homogenized sample to measure the fluorescence signal was not as predictive as whole-body cortisol and eGFP mRNA measurements to assess stress-response. Improving the extraction method, enhancing protein integrity preservation, optimizing larval sampling protocols, and employing different imaging instruments or techniques (e.g., confocal measurement) may improve the sensitivity and predictive value of eGFP protein. To assess stress disruption.

The major obstacles in the design and development of environmental risk assessment surveillance tools are defining exposure settings (acute vs chronic), appropriate biomarkers, ideal tissue specimens, ability to detect the effect of a single or combinations of toxicants, ability to screen for potential within-generational and transgenerational effects, and finally the associated cost and volume capacity of screening an array of compounds (4). Using transgenic zebrafish larvae, such as SR4G, could address many of these issues. For example, we showed that predictive models can be created to detect a physiological response using the transgene alone, or in combination with other genes of interest. Additionally, the proposed approach has the potential to discriminate between different exposure settings (e.g., chronic vs acute). Using NPV calculation we showed that the proposed approach could detect a disrupted stressed response for some chemicals. More testing with a wider of potential EDCs is required to validate this model further. We believe this approach can potentially be adapted to detect the effects of many other EDCs by introducing different transgenic constructs.

## Data Availability Statement

The original contributions presented in the study are included in the article/**Supplementary Material**. Further inquiries can be directed to the corresponding author.

## Ethics Statement

The animal study was reviewed and approved by Animal Care and Veterinary Services (ACVS) of the University of Ottawa.

## Author Contributions

AN and VT designed the study, developed the methodology, conducted the experiments, and prepared the manuscript. SD conducted experiments and helped with the maintenance of the zebrafish line. All authors contributed to the article and approved the submitted version.

## Funding

The research was funded by the NSERC Discovery Grant Programs and the University of Ottawa Research Chair (2016-21) in Neuroendocrinology (VT).

## Conflict of Interest

The authors declare that the research was conducted in the absence of any commercial or financial relationships that could be construed as a potential conflict of interest.

## Publisher’s Note

All claims expressed in this article are solely those of the authors and do not necessarily represent those of their affiliated organizations, or those of the publisher, the editors and the reviewers. Any product that may be evaluated in this article, or claim that may be made by its manufacturer, is not guaranteed or endorsed by the publisher.

## Supplementary Material

Supplementary Figure 1 Whole-body cortisol and eGFP mRNA levels in 7dpf transgenic zebrafish larvae exposed to cortisol. The cortisol levels are measured in pg/larva and normalized to the control unstressed condition. Panel **(A)** shows the whole-body cortisol in the cortisol treated group (CORT) with its corresponding control group (DMSO). The white bars represent cortisol levels in the unstressed condition (sacrificing subjects at 0 minutes before the handling stress) and the black bars represent cortisol levels 30 minutes after the handling stress. CORT: overnight exposure to 10µM cortisol; DMSO: overnight exposure to DMSO; 0 min: unstressed; 30 min: 30 minutes post handling stress. DMSO was used as the vehicle in a final concentration of 0.001%. Two-way ANOVA was performed. Tukey’s post-hoc test followed the significant ANOVA results for pairwise comparisons. Means (± SEM) with different letters (a, b) are significantly different; p≤0.05; N=6. Each sample contained a pool of 22-28 7dpf SR4G transgenic zebrafish larvae. Panel **(B)** shows the eGFP mRNA levels in cortisol (CORT) treated groups with its corresponding control group (DMSO) following overnight exposure to cortisol. The mRNA levels are measured in normalized fold change compared to the control unstressed condition. The white bars represent eGFP mRNA levels in the unstressed condition (sacrificing subjects at 0 minutes before the handling stress) and the gray bars represent eGFP mRNA levels 60 minutes after the final round of handling stress. CORT: overnight exposure to cortisol 10µM; DMSO: overnight exposure to DMSO; 0 min: unstressed; 60 min: 60 minutes post handling stress. DMSO was used as the vehicle in a final concentration of 0.001%. Two-way ANOVA was performed. Means (± SEM) with an asterisk (*) are significantly different compared to the control unstressed state (p≤0.05); N=6. Each sample contained a pool of 22-28 7dpf SR4G transgenic zebrafish larvae.

Supplementary Figure 2 The whole-body mRNA levels of genes related to stress, depression, and neurogenesis following exposure to FLX, BPA, VIN, and DEX. The 9 genes with similar fold change ranges are separated from bdnf and fosab for visual illustration purposes. The white and black bars show unstressed and stressed conditions, respectively. In each treatment and control groups the 9 genes are arranged alphabetically; from left to right: 1:crhb, 2:egr2a, 3:fkbp5, 4:fosl1a, 5:htr1b, 6:npas4a, 7:nr4a1, 8:per2, and 9:rorcb. From top to bottom graphs show the mRNA levels of the 9 genes in the FLX-O/N, FLX-6D, BPA, VIN, and DEX groups and their related control group. FLX-O/N: overnight exposure to fluoxetine; FLX-6D: daily exposure to fluoxetine from 0 to 6 days post fertilization(dpf); BPA: overnight exposure to bisphenol A; VIN: overnight exposure to vinclozolin; DEX: overnight exposure to dexamethasone; Ethanol: overnight exposure to ethanol, Ethanol6D: daily exposure to ethanol from 0dpf to 6dpf; DMSO: overnight exposure to DMSO. Ethanol was used as the vehicle for FLX. Ethanol final concentrations in all experiments was 0.005%. DMSO was used as the vehicle for BPA, VIN, and DEX experiments in a final concentration of 0.001%. Two-way ANOVA was performed for each gene in each treatment group and its related control group in both unstressed and stressed state. No statistical comparison was performed between different genes or different treatment groups. If the ANOVA for treatment, stress state and their interaction were significant (p ≤0.05), Tukey’s post-hoc test was performed for all conditions. The means (± SEM) with different letters (a, b) are significantly different; p≤0.05; N=6. If the ANOVA for treatment and stress state was significant (p≤0.05) and ANOVA for interaction between them (treatment vs stress state) was not significant (intercept p >0.05), Tukey’s post-hoc test was only performed for stress state within each treatment group. The means (± SEM) with a significant difference is marked with an asterisk (*) in this case; p≤0.05; N=6. Each sample was a pool of 22-28 7dpf SR4G transgenic zebrafish larvae.

Supplementary Figure 3 The whole-body mRNA levels of bdnf and fosab related to stress, depression, and neurogenesis following exposure to FLX, BPA, VIN, and DEX. The white and black bars show unstressed and stressed conditions, respectively. Panel **(A)** shows whole-body mRNA levels for bdnf. From top to bottom graphs show bndf mRNA levels in the FLX-O/N, FLX-6D, BPA, VIN, and DEX groups and their related control group. Panel **(B)** shows whole-body mRNA levels for fosab. From top to bottom graphs show fosab mRNA levels in the FLX-O/N, FLX-6D, BPA, VIN, and DEX groups and their related control group. FLX-O/N: overnight exposure to fluoxetine; FLX-6D: daily exposure to fluoxetine from 0 to 6 days post fertilization(dpf); BPA: overnight exposure to bisphenol A; VIN: overnight exposure to vinclozolin; DEX: overnight exposure to dexamethasone; Ethanol: overnight exposure to ethanol, Ethanol6D: daily exposure to ethanol from 0dpf to 6dpf; DMSO: overnight exposure to DMSO. Ethanol was used as the vehicle for FLX. Ethanol final concentrations in all experiments was 0.005%. DMSO was used as the vehicle for BPA, VIN, and DEX experiments in a final concentration of 0.001%. Two-way ANOVA was performed for each gene in each treatment group and its related control group in both unstressed and stressed state. No statistical comparison was performed between different genes or different treatment groups. If the ANOVA for treatment, stress state and their interaction were significant (p ≤0.05), Tukey’s post-hoc test was performed for all conditions. The means (± SEM) with different letters (a, b) are significantly different; p≤0.05; N=6. If the ANOVA for treatment and stress state was significant (p≤0.05) and ANOVA for interaction between them (treatment vs stress state) was not significant (intercept p >0.05), Tukey’s post-hoc test was only performed for stress state within each treatment group. The means (± SEM) with a significant difference is marked with an asterisk (*) in this case; p≤0.05; N=6. Each sample was a pool of 22-28 7dpf SR4G transgenic zebrafish larvae.

Supplementary Figure 4 The whole-body fluorescence intensity in the crude homogenized extract of following exposure to different chemicals. The fluorescence values are measured in fluorescence intensity unit (FIU)/mg of total protein and normalized to the control unstressed condition. The graphs from top to bottom show the whole-body fluorescence intensity measurement following exposure to FLX-O/N, FLX-6D, BPA, VIN, and DEX. The white bars represent fluorescence intensity levels in the unstressed condition (sacrificing subjects at 0 minutes before the handling stress) and the black bars represent fluorescence intensity levels 120 minutes after the final round of handling stress. FLX-O/N: overnight exposure to fluoxetine; FLX-6D: daily exposure to fluoxetine from 0 to 6 days post fertilization(dpf); BPA: overnight exposure to bisphenol A; VIN: overnight exposure to vinclozolin; DEX: overnight exposure to dexamethasone, Ethanol-O/N: overnight exposure to ethanol, Ethanol-6D: daily exposure to ethanol from 0dpf to 6dpf; 0 min: unstressed; 120 min: 120 minutes post handling stress; DMSO: overnight exposure to DMSO; 0 min: unstressed; 120 min: 120 minutes post handling stress. Ethanol final concentrations in all experiments was 0.005%. DMSO was used as the vehicle for BPA, VIN, and DEX experiments in a final concentration of 0.001%. The DMSO data in each graph is the same data portrayed for comparison purposes. Two-way ANOVA was performed between each treatment group and its related control group in both unstressed and stressed state. No statistical comparison was performed between different treatment groups. The ANOVA results were not significant across all treatment and control groups; p>0.05; N=6. Each sample contained 22-28, 7dpf SR4G transgenic zebrafish larvae.

Supplementary Figure 5 The correlation between whole-body cortisol and total eGFP mRNA levels in SR4G transgenic zebrafish larvae. The graphs in panel **(A)** show normalized whole-body cortisol levels (left) and normalized whole-body eGFP mRNA levels (right) in the two control groups, Ethanol and DMSO, under the two unstressed and stressed conditions. Two-way ANOVA was performed to evaluate the effect of treatments and stress conditions on whole-body cortisol levels and whole-body eGFP mRNA levels. Means (± SEM) marked by an asterisk (*) are significantly different within each category (whole-body cortisol or whole-body eGFP mRNA study); p≤0.0001; N=6. Ethanol: overnight exposure to 0.005% ethanol; DMSO: overnight exposure to 0.001% DMSO. The time points,30 min or 60 min, show the time-lapsed following the handling stress before euthanizing the subjects for sample collection. Each sample contained a pool of 7dpf SR4G transgenic zebrafish larvae(n=22-28). Panel **(B)** shows the correlation coefficient between whole-body cortisol and whole-body eGFP mRNA illustrated by graphs and determined by the Pearson test (r=0.947).
