# Supplementary figures and images for: Applications of the SR4G Transgenic Zebrafish Line for Biomonitoring of Stress-Disrupting Compounds: A Proof-of-Concept Study
Source: Front Endocrinol (Lausanne). 2021 Nov 17;12:727777. doi: 10.3389/fendo.2021.727777 (PMC8635770; doi:10.3389/fendo.2021.727777)

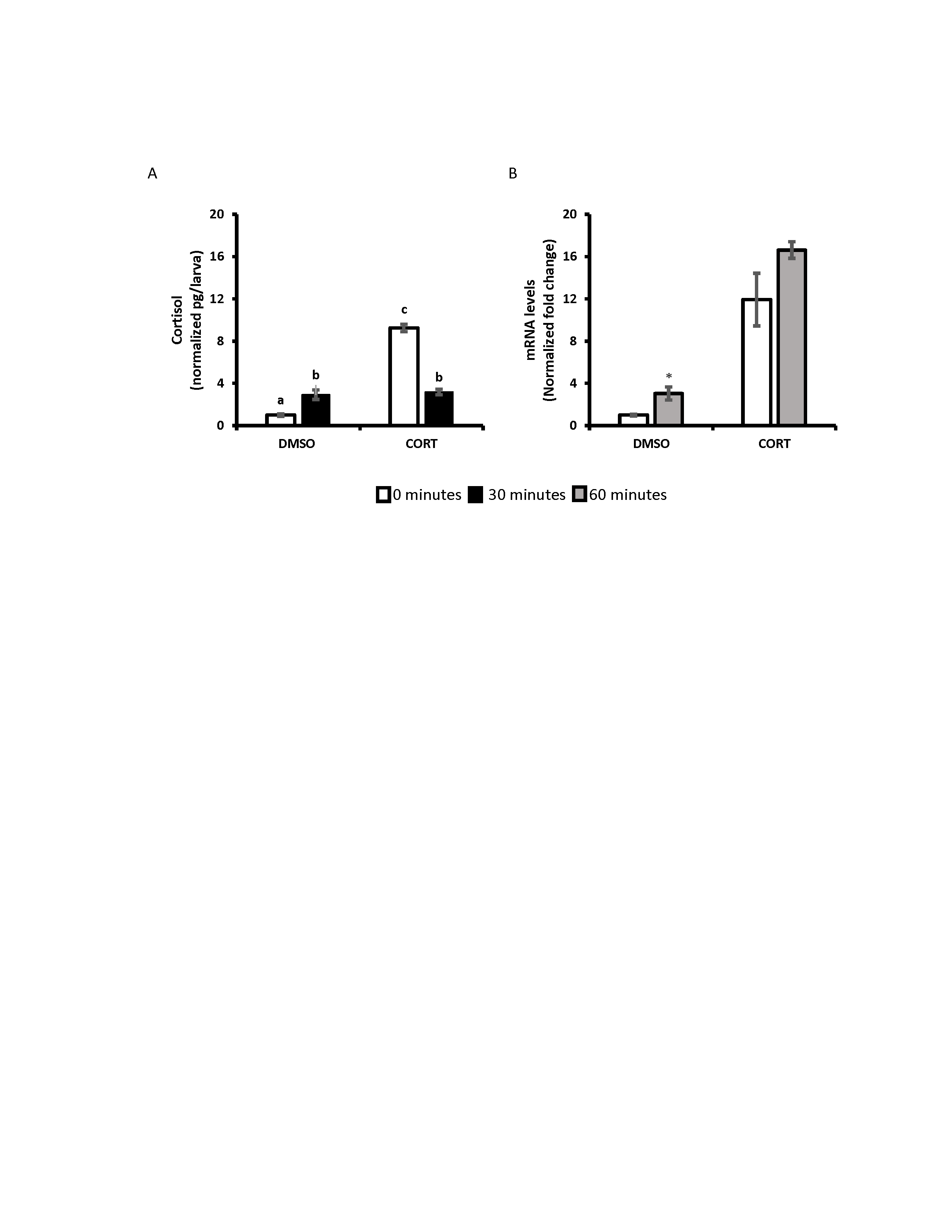

Supplement: Supplementary Figure 1 — Whole-body cortisol and eGFP mRNA levels in 7dpf transgenic zebrafish larvae exposed to cortisol. The cortisol levels are measured in pg/larva and normalized to the control unstressed condition. Panel (A) shows the whole-body cortisol in the cortisol treated group (CORT) with its corresponding control group (DMSO). The white bars represent cortisol levels in the unstressed condition (sacrificing subjects at 0 minutes before the handling stress) and the black bars represent cortisol levels 30 minutes after the handling stress. CORT: overnight exposure to 10µM cortisol; DMSO: overnight exposure to DMSO; 0 min: unstressed; 30 min: 30 minutes post handling stress. DMSO was used as the vehicle in a final concentration of 0.001%. Two-way ANOVA was performed. Tukey’s post-hoc test followed the significant ANOVA results for pairwise comparisons. Means (± SEM) with different letters (a, b) are significantly different; p≤0.05; N=6. Each sample contained a pool of 22-28 7dpf SR4G transgenic zebrafish larvae. Panel (B) shows the eGFP mRNA levels in cortisol (CORT) treated groups with its corresponding control group (DMSO) following overnight exposure to cortisol. The mRNA levels are measured in normalized fold change compared to the control unstressed condition. The white bars represent eGFP mRNA levels in the unstressed condition (sacrificing subjects at 0 minutes before the handling stress) and the gray bars represent eGFP mRNA levels 60 minutes after the final round of handling stress. CORT: overnight exposure to cortisol 10µM; DMSO: overnight exposure to DMSO; 0 min: unstressed; 60 min: 60 minutes post handling stress. DMSO was used as the vehicle in a final concentration of 0.001%. Two-way ANOVA was performed. Means (± SEM) with an asterisk (*) are significantly different compared to the control unstressed state (p≤0.05); N=6. Each sample contained a pool of 22-28 7dpf SR4G transgenic zebrafish larvae. [file Image_1.tif]

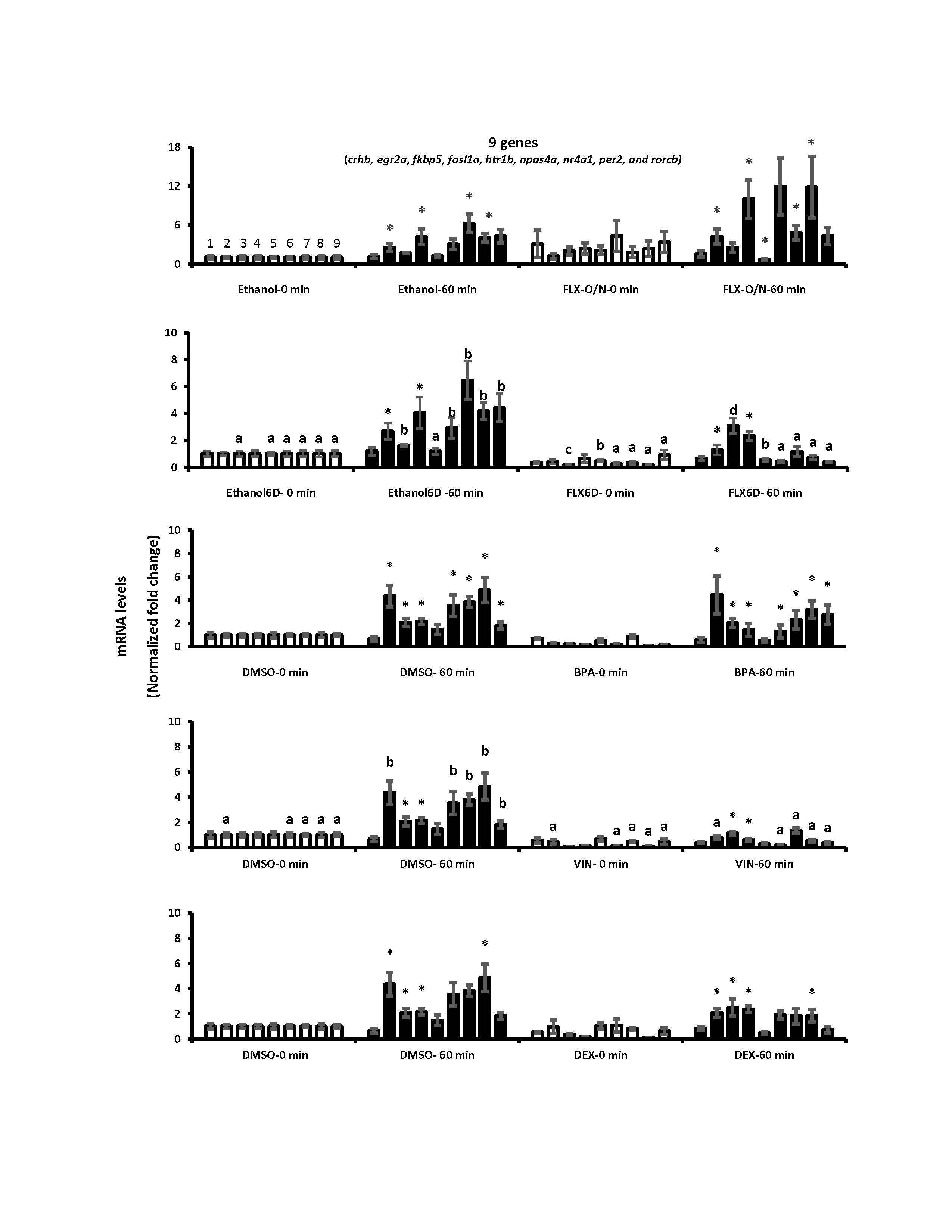

Supplement: Supplementary Figure 2 — The whole-body mRNA levels of genes related to stress, depression, and neurogenesis following exposure to FLX, BPA, VIN, and DEX. The 9 genes with similar fold change ranges are separated from bdnf and fosab for visual illustration purposes. The white and black bars show unstressed and stressed conditions, respectively. In each treatment and control groups the 9 genes are arranged alphabetically; from left to right: 1:crhb, 2:egr2a, 3:fkbp5, 4:fosl1a, 5:htr1b, 6:npas4a, 7:nr4a1, 8:per2, and 9:rorcb. From top to bottom graphs show the mRNA levels of the 9 genes in the FLX-O/N, FLX-6D, BPA, VIN, and DEX groups and their related control group. FLX-O/N: overnight exposure to fluoxetine; FLX-6D: daily exposure to fluoxetine from 0 to 6 days post fertilization(dpf); BPA: overnight exposure to bisphenol A; VIN: overnight exposure to vinclozolin; DEX: overnight exposure to dexamethasone; Ethanol: overnight exposure to ethanol, Ethanol6D: daily exposure to ethanol from 0dpf to 6dpf; DMSO: overnight exposure to DMSO. Ethanol was used as the vehicle for FLX. Ethanol final concentrations in all experiments was 0.005%. DMSO was used as the vehicle for BPA, VIN, and DEX experiments in a final concentration of 0.001%. Two-way ANOVA was performed for each gene in each treatment group and its related control group in both unstressed and stressed state. No statistical comparison was performed between different genes or different treatment groups. If the ANOVA for treatment, stress state and their interaction were significant (p ≤0.05), Tukey’s post-hoc test was performed for all conditions. The means (± SEM) with different letters (a, b) are significantly different; p≤0.05; N=6. If the ANOVA for treatment and stress state was significant (p≤0.05) and ANOVA for interaction between them (treatment vs stress state) was not significant (intercept p >0.05), Tukey’s post-hoc test was only performed for stress state within each treatment group. The means (± SEM) with a significant differenc [file Image_2.tif]

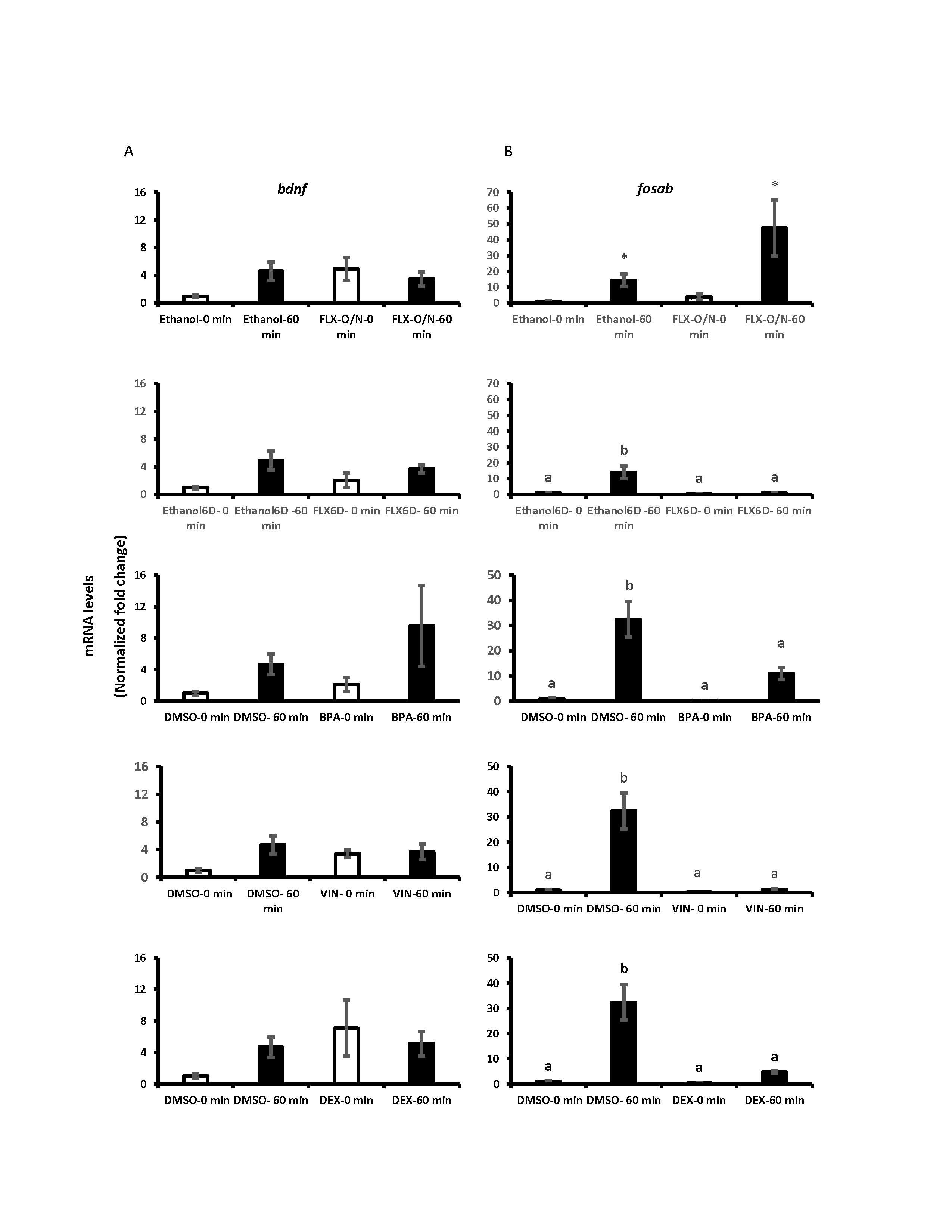

Supplement: Supplementary Figure 3 — The whole-body mRNA levels of bdnf and fosab related to stress, depression, and neurogenesis following exposure to FLX, BPA, VIN, and DEX. The white and black bars show unstressed and stressed conditions, respectively. Panel (A) shows whole-body mRNA levels for bdnf. From top to bottom graphs show bndf mRNA levels in the FLX-O/N, FLX-6D, BPA, VIN, and DEX groups and their related control group. Panel (B) shows whole-body mRNA levels for fosab. From top to bottom graphs show fosab mRNA levels in the FLX-O/N, FLX-6D, BPA, VIN, and DEX groups and their related control group. FLX-O/N: overnight exposure to fluoxetine; FLX-6D: daily exposure to fluoxetine from 0 to 6 days post fertilization(dpf); BPA: overnight exposure to bisphenol A; VIN: overnight exposure to vinclozolin; DEX: overnight exposure to dexamethasone; Ethanol: overnight exposure to ethanol, Ethanol6D: daily exposure to ethanol from 0dpf to 6dpf; DMSO: overnight exposure to DMSO. Ethanol was used as the vehicle for FLX. Ethanol final concentrations in all experiments was 0.005%. DMSO was used as the vehicle for BPA, VIN, and DEX experiments in a final concentration of 0.001%. Two-way ANOVA was performed for each gene in each treatment group and its related control group in both unstressed and stressed state. No statistical comparison was performed between different genes or different treatment groups. If the ANOVA for treatment, stress state and their interaction were significant (p ≤0.05), Tukey’s post-hoc test was performed for all conditions. The means (± SEM) with different letters (a, b) are significantly different; p≤0.05; N=6. If the ANOVA for treatment and stress state was significant (p≤0.05) and ANOVA for interaction between them (treatment vs stress state) was not significant (intercept p >0.05), Tukey’s post-hoc test was only performed for stress state within each treatment group. The means (± SEM) with a significant difference is marked with an asterisk (*) in this case; p≤0.05; N=6. Each sampl [file Image_3.tif]

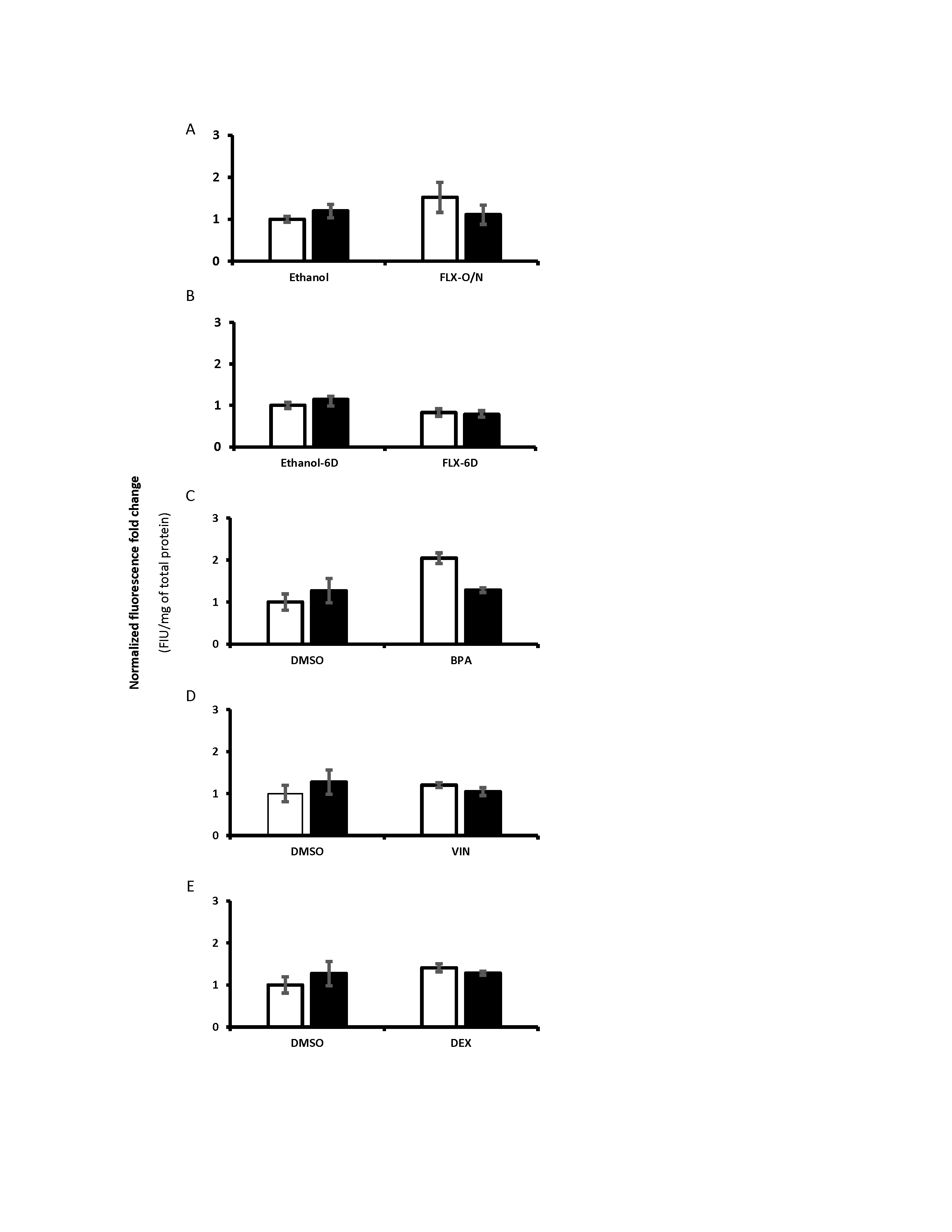

Supplement: Supplementary Figure 4 — The whole-body fluorescence intensity in the crude homogenized extract of following exposure to different chemicals. The fluorescence values are measured in fluorescence intensity unit (FIU)/mg of total protein and normalized to the control unstressed condition. The graphs from top to bottom show the whole-body fluorescence intensity measurement following exposure to FLX-O/N, FLX-6D, BPA, VIN, and DEX. The white bars represent fluorescence intensity levels in the unstressed condition (sacrificing subjects at 0 minutes before the handling stress) and the black bars represent fluorescence intensity levels 120 minutes after the final round of handling stress. FLX-O/N: overnight exposure to fluoxetine; FLX-6D: daily exposure to fluoxetine from 0 to 6 days post fertilization(dpf); BPA: overnight exposure to bisphenol A; VIN: overnight exposure to vinclozolin; DEX: overnight exposure to dexamethasone, Ethanol-O/N: overnight exposure to ethanol, Ethanol-6D: daily exposure to ethanol from 0dpf to 6dpf; 0 min: unstressed; 120 min: 120 minutes post handling stress; DMSO: overnight exposure to DMSO; 0 min: unstressed; 120 min: 120 minutes post handling stress. Ethanol final concentrations in all experiments was 0.005%. DMSO was used as the vehicle for BPA, VIN, and DEX experiments in a final concentration of 0.001%. The DMSO data in each graph is the same data portrayed for comparison purposes. Two-way ANOVA was performed between each treatment group and its related control group in both unstressed and stressed state. No statistical comparison was performed between different treatment groups. The ANOVA results were not significant across all treatment and control groups; p>0.05; N=6. Each sample contained 22-28, 7dpf SR4G transgenic zebrafish larvae. [file Image_4.tif]

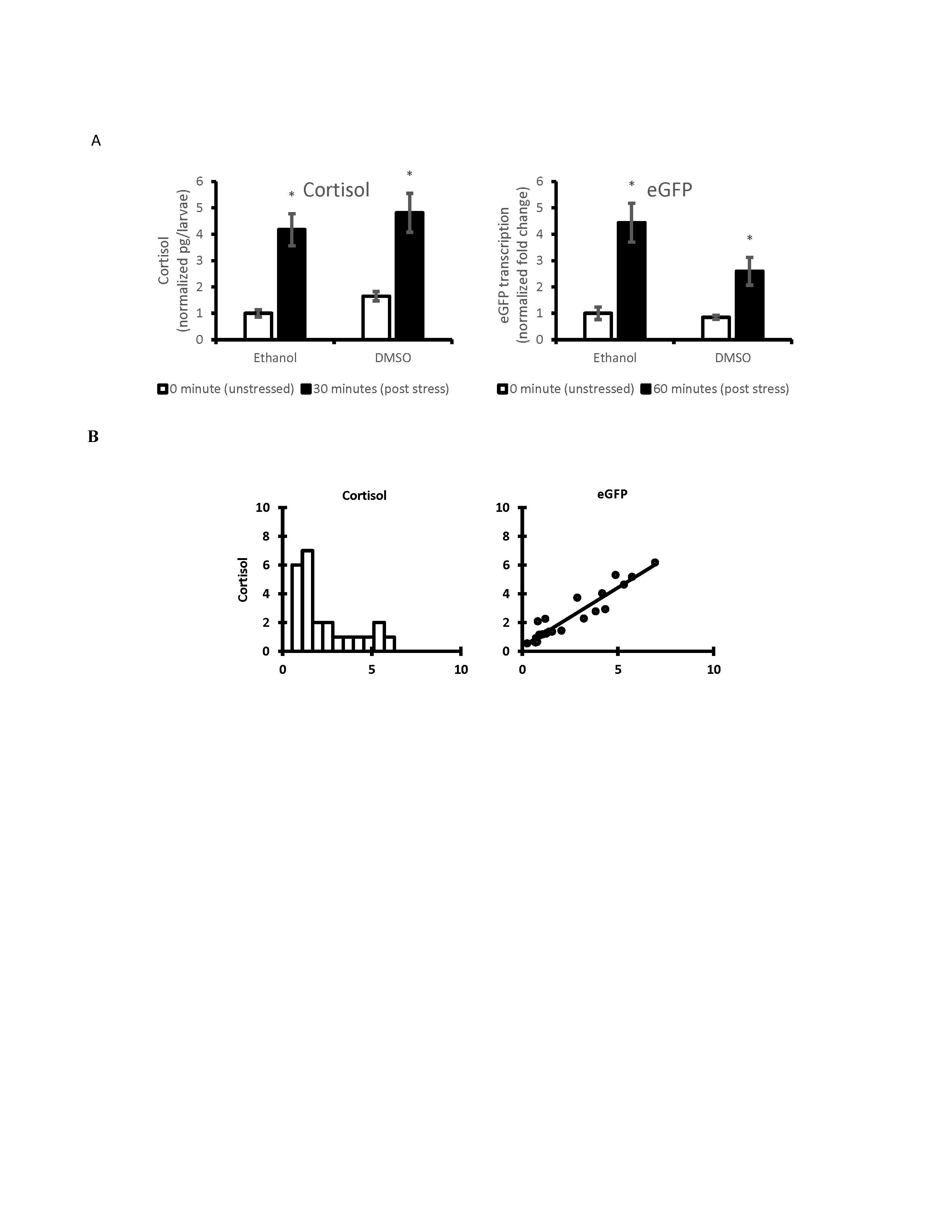

Supplement: Supplementary Figure 5 — The correlation between whole-body cortisol and total eGFP mRNA levels in SR4G transgenic zebrafish larvae. The graphs in panel (A) show normalized whole-body cortisol levels (left) and normalized whole-body eGFP mRNA levels (right) in the two control groups, Ethanol and DMSO, under the two unstressed and stressed conditions. Two-way ANOVA was performed to evaluate the effect of treatments and stress conditions on whole-body cortisol levels and whole-body eGFP mRNA levels. Means (± SEM) marked by an asterisk (*) are significantly different within each category (whole-body cortisol or whole-body eGFP mRNA study); p≤0.0001; N=6. Ethanol: overnight exposure to 0.005% ethanol; DMSO: overnight exposure to 0.001% DMSO. The time points,30 min or 60 min, show the time-lapsed following the handling stress before euthanizing the subjects for sample collection. Each sample contained a pool of 7dpf SR4G transgenic zebrafish larvae(n=22-28). Panel (B) shows the correlation coefficient between whole-body cortisol and whole-body eGFP mRNA illustrated by graphs and determined by the Pearson test (r=0.947). [file Image_5.tif]
