# Supplementary material for: Applications of the SR4G Transgenic Zebrafish Line for Biomonitoring of Stress-Disrupting Compounds: A Proof-of-Concept Study
Source: Front Endocrinol (Lausanne). 2021 Nov 17;12:727777. doi: 10.3389/fendo.2021.727777 (PMC8635770; doi:10.3389/fendo.2021.727777)
Supplement: Supplementary file 6 [file Table_1.pdf]

Supplementary table 1. Prediction of stress response based on the targeted genes' mRNA levels using regression random forest (RDF) and logistic regression (LOG) model trained by Ethanol (control) group.

Ethanol (control)	Predicted stress status											
	FLX_O/N		FLX-6D		DMSO		BPA		VIN		DEX	
Samples	LOG	RDF	LOG	RDF	LOG	RDF	LOG	RDF	LOG	RDF	LOG	RDF
Uns-1	0	1	0	0	0	0	0	0	0	0	0	0
Uns-2	1	1	0	0	0	0	0	0	0	0	0	0
Uns-3	0	0	0	0	0	0	0	0	0	0	0	0
Uns-4	0	0	0	0	0	0	0	0	0	0	0	0
Uns-5	1	0	0	0	0	0	0	0	0	0	0	0
Uns-6	1	1	0	0	0	0	0	0	0	0	0	1
St-1	1	1	0	1	1	1	1	1	0	0	0	1
St-2	1	1	1	1	1	1	1	1	0	0	0	1
St-3	1	1	1	0	1	1	0	1	1	0	0	1
St-4	0	0	0	0	1	1	1	1	0	0	0	1
St-5	1	1	0	1	1	1	1	1	0	0	0	1
St-6	1	1	1	1	1	1	1	1	1	0	0	1
**PPV**	**62%**	**62%**	N/A	N/A	**100%**	**100%**	N/A	N/A	N/A	N/A	N/A	N/A
**NPV**	**75%**	**75%**	**100%**	**100%**	**100%**	**100%**	**100%**	**100%**	**100%**	**100%**	**100%**	**100%**

FLX-6D: daily exposure to from 0 to 6 days post fertilization(dpf); FLX-O/N, BPA, VIN, DEX, CORT representing overnight exposure to fluoxetine, bis-phenol A, vinclozolin, dexamethasone, and cortisol, respectively. LOG: logistic regression, RFD: random forest. PPV: positive predictive value, NPV: negative predictive value. Uns: unstressed sample; St: stressed sample. (LOG; R^2: 0.774) (RDF; OOB: 0.4)
