# Supplementary material for: Applications of the SR4G Transgenic Zebrafish Line for Biomonitoring of Stress-Disrupting Compounds: A Proof-of-Concept Study
Source: Front Endocrinol (Lausanne). 2021 Nov 17;12:727777. doi: 10.3389/fendo.2021.727777 (PMC8635770; doi:10.3389/fendo.2021.727777)
Supplement: Supplementary file 7 [file Table_2.pdf]

Supplementary table 2. The primers list for qPCR experiments

**Gene symbol**	**Primer sequence**	
Danio rerio	Forward primer 5' to 3'	Forward primer 5' to 3'
eef1a1l1	CCG TCT GCC ACT TCA GGA TGT GT	TTG AGG ACA CCA GTC TCC ACA CGA
d4EGFP	CGA GCA ACT GAG GAT CCC ATT CTC T	CAC CCC GGT GAA CAG CTC CT
bdnf	AGC ATC TGT TGG AGT GTG TGG	TAA CCT GTT GGA ACA TTT TCC CCT
crhb	CAC AGA TTC TCC TCG CCA CT	TGG AAA GGC AAC GAG CAG AG
egr2a	CAA CAC AAG CCC TCA GAG CAA	GTC GCT GTC ATT TTG ATC CTC G
fkbp5	CGC CGG TGA GAC TAA ACA GA	ACA TGC CCT TGT TCC CAA AA
fosab	TAC CCG CTC AAC CAG ACT CA	CGT GAC AGT TGG CAC GAA AG
fosl1a	CCC TGA CTC CAT TTA CCG CC	GAC GGA TGA GAC GTG ACG AG
htr1b	TTC CCC TCT GTT CGT CTT GC	TGA GTT GAC GTA GCC AAG CC
npas4a	GGT CCA CTA AAG GAG CCT CG	TCG GAG ATG GGC AAC AAG TC
nr4a1	TCG CTT ACG GTT TCT CTG CT	AGG TCT TCA AAC ACA GGC GT
per2	GAG AGG GGT CCA CGC TTT TA	GGG ACT GCT TCA GAC GTG AC
rorcb	ACC TGT CAA CAT GAG AGC CC	CCT TGC ACC CTT CAC ACG TA
