# Supplementary material for: Applications of the SR4G Transgenic Zebrafish Line for Biomonitoring of Stress-Disrupting Compounds: A Proof-of-Concept Study
Source: Front Endocrinol (Lausanne). 2021 Nov 17;12:727777. doi: 10.3389/fendo.2021.727777 (PMC8635770; doi:10.3389/fendo.2021.727777)
Supplement: Supplementary file 8 [file Table_3.pdf]

Supplementary table 3. The 11 genes implicated in stress response, neural plasticity, and development of depression based on *Homo sapiens* orthologue pathways.

Gene Symbol		Gene name	Homo sapiens orthologue pathway
Danio rerio	***Homo sapiens***		
d4EGFP	NA	transgene-Green Fluorescent Protein	NA
bdnf	BDNF	Brain-derived neurotrophic factor	Development of depression, Stress, Neural plasticity
crhb	CRH	corticotropin releasing hormone b	Stress response
egr2a	EGR⁺	early growth response 2a	Neural plasticity
fkbp5	FKBP5	FKBP Prolyl Isomerase 5	Development of depression, Stress response
fosab	FOS	v-fos FBJ murine osteosarcoma viral oncogene homolog Ab	Development of depression, Neural plasticity
fosl1a	FOSL1	FOS Like 1, AP-1 Transcription Factor Subunit	Development of depression
htr1b	HTR1D	5-hydroxytryptamine (serotonin) receptor 1B	Development of depression
npas4a	NPAS4	neuronal PAS domain protein 4a	Neural plasticity
nr4a1	NR4A1	nuclear receptor subfamily 4, group A	Stress response
per2	PER2	period circadian clock 2	Stress response, Neural plasticity
rorcb	RORC⁺	RAR-related orphan receptor C b	Stress response

NA: not applicable; * Indicates a gene that did not pass FDR≤ 0.05; (+): potential orthologue
